# MgrB Alterations Mediate Colistin Resistance in *Klebsiella pneumoniae* Isolates from Iran

**DOI:** 10.3389/fmicb.2017.02470

**Published:** 2017-12-18

**Authors:** Mehri Haeili, Afsaneh Javani, Jale Moradi, Zeinab Jafari, Mohammad M. Feizabadi, Esmaeil Babaei

**Affiliations:** ^1^Department of Biology, Faculty of Natural Sciences, University of Tabriz, Tabriz, Iran; ^2^Department of Microbiology, School of Medicine, Kermanshah University of Medical Sciences, Kermanshah, Iran; ^3^Department of Microbiology, School of Medicine, Tehran University of Medical Sciences, Tehran, Iran; ^4^Thorax Research Center, Imam Khomeini Hospital, Tehran University of Medical Sciences, Tehran, Iran

**Keywords:** colistin resistance, *Klebsiella pneumoniae*, PmrAB, MgrB, PhoPQ

## Abstract

Colistin is one of the last-resort therapeutic agents to combat multidrug-resistant Gram-negative bacteria (GNB) including *Klebsiella pneumoniae*. Although it happens rarely, resistance to colistin has been reported for several GNB. A total of 20 colistin resistant (col-R) and three colistin susceptible (col-S) clinical isolates of *K. pneumoniae* were studied to explore the underlying mechanisms of colistin resistance. The presence of plasmid encoded resistance genes, *mcr-1, mcr-2, mcr-3, and mcr-4* genes were examined by PCR. The nucleotide sequences of *pmrA, pmrB, phoP, phoQ*, and *mgrB* genes were determined. To evaluate the association between colistin resistance and upregulation of *pmrHFIJKLM* and *pmrCAB* operons, transcriptional level of the *pmrK* and *pmrC* genes encoding for lipopolysaccharide target modifying enzymes was quantified by RT-qPCR analysis. None of the plasmid encoded resistance genes were detected in the studied isolates. Inactivation of MgrB due to nonsense mutations and insertion of IS elements was observed in 15 col-R isolates (75%). IS elements (IS*5*-like and IS*1*-like families) most commonly targeted the coding region and in one case the promoter region of the *mgrB.* Complementation with wild-type MgrB restored colistin susceptibility in isolates with altered *mgrB*. All col-R isolates lacked any genetic alterations in the *pmrA, phoP*, and *phoQ* genes and substitutions identified in the *pmrB* were not found to be involved in resistance conferring determined by complementation assay. Colistin resistance linked with upregulation of *pmrHFIJKLM* and *pmrCAB* operons with the *pmrK* and *pmrC* being overexpressed in 20 and 11 col-R isolates, respectively. Our results demonstrated that MgrB alterations are the major mechanisms contributing to colistin resistance in the tested *K. pneumoniae* isolates from Iran.

## Introduction

The emergence of MDR *Enterobacteriaceae* and the lack of new antibiotics coming to market to combat them bring us perilously to the end of the “Antibiotic era.” Among *Enterobacteriaceae, Klebsiella pneumoniae*, is found to be associated with the highest rates of carbapenem resistance (Marinelli and Genilloud, 2013). Polymyxin antibiotics such as colistin are among the few antimicrobial agents that retain activity against MDR-GNB in particular CRE and often considered as the last-line therapy to treat infections caused by these superbugs (Li et al., 2006; Olaitan et al., 2014b). Polymyxins (polymyxin B and colistin) are cationic antimicrobial peptides that target anionic lipid A phosphate moiety of bacterial LPS causing membrane leakage and eventually cell death (Gupta et al., 2009; Deris et al., 2014). Bacteria employ several means to protect themselves from antimicrobial activity of polymyxins. The most common resistance mechanism in *Enterobacteriaceae* is attributed to the covalent modifications of the lipid A moiety of LPS through the incorporation of positively charged groups such as PEtN and L-Ara4N. These modifications neutralize the negative charges of LPS and subsequently reduce binding affinity of colistin to its target (Tamayo et al., 2005). LPS modification in *K. pneumoniae* is mediated by the activation of PmrA/PmrB two-component regulatory system (TCRS) which regulates expression of the genes located in *pmrCAB* and *pmrHFIJKLM* operons encoding for enzymes implicated in the LPS modifications. Moreover, a second TCRS, PhoP/PhoQ is known to contribute to polymyxin resistance by indirectly activating the PmrA/PmrB TCRS via PmrD linker protein. Mutations in these TCRSs can cause constitutive expression of the *pmrCAB* and *pmrHFIJKLM* operons and consequently transfer of PEtN and L-Ara4N, respectively, to lipid A (Tamayo et al., 2005; Olaitan et al., 2014b). Moreover, inactivation of the PhoQ/PhoP negative regulator encoded by *mgrB* gene has been identified to play a prominent role in polymyxin resistance in *K. pneumoniae* isolates (Cannatelli et al., 2014b; Olaitan et al., 2014b; Poirel et al., 2015). Genetic alterations such as Leu26Pro in PhoQ (Cheng et al., 2015), Leu82Arg (Cannatelli et al., 2014a), and Thr157Pro (Jayol et al., 2014) in PmrB, Q30stop, C28stop and insertion of IS elements in MgrB (Poirel et al., 2015) have been found to contribute to colistin resistance in *K. pneumoniae* isolates. In addition to these chromosomally mediated mechanisms, plasmid-encoded colistin resistance genes, *mcr-1, mcr-2, mcr-3, and mcr-4* encoding a PEtN transferase have been recently reported as a transmissible resistance mechanism in *Enterobacteriaceae* (Gu et al., 2016; Liu et al., 2016; Xavier et al., 2016; Carattoli et al., 2017; Yin et al., 2017). Due to lack of information about the molecular mechanisms of colistin resistance in *K. pneumoniae* isolates in Iran, we aimed to explore these mechanisms in colistin resistant *K. pneumoniae* strains from this geographic region.

## Materials and Methods

### Bacterial Strains and Antimicrobial Susceptibility Testing

A total of 20 col-R KP clinical strains isolated from the same number of patients hospitalized in Imam Khomeini hospital, the largest medical center of the country with 1,400 beds were studied. The isolates were obtained between 2015 and 2017 through antimicrobial resistance surveillance study for detection of col-R isolates. MICs of colistin were determined by broth macrodilution method using colistin sulfate (Sigma–Aldrich) over a range of dilutions from 0.125 to 128 μg/ml according to instructions described by Wiegand et al. (2008) and Clinical and Laboratory Standards Institute (2014). Isolates characterized with MIC values greater than 2 μg/ml were categorized as resistant according to guidelines described by the European Committee on Antimicrobial Susceptibility Testing (EUCAST)^1^. *K. pneumoniae* ATCC 700603 was used as a quality control for antimicrobial susceptibility testing.

### Bacterial Genotyping

The genetic relatedness of the col-R KP isolates was assessed by pulsed-field gel electrophoresis (PFGE) analysis using *Xba*I digested genomic DNA as described previously (Ribot et al., 2006) and results were interpreted as recommended by Tenover et al. (1995).

### Molecular Characterization of Colistin Resistance

In order to investigate the possible contribution of plasmid encoded *mcr-1, mcr-2, mcr-3*, and *mcr-4* genes in resistance development, genomic DNAs were obtained from the isolates and were subjected to polymerase chain reaction (PCR) using previously described primers (Liu et al., 2016; Xavier et al., 2016; Carattoli et al., 2017; Yin et al., 2017) (**Table 1**). To detect any genetic alterations associated with colistin resistance, *pmrA, pmrB, phoP, phoQ*, and *mgrB* genes were amplified using primers listed in **Table 1**. The resulting amplicons were sent to SEQLAB Sequence Laboratories (Göttingen GmbH, Germany) for sequencing. Genomic DNA from *K. pneumoniae* ATCC 700603 and three other col-S *K. pneumoniae* clinical isolates were used as control.

**Table 1:** Nucleotide sequences of primers used in this study.

Primer name	Sequence (5′ to 3′)	Size of product (bp)	Reference
Sequencing			
pmrA-F	CGCAGGATAATCTGTTCTCCA	808	This study
pmrA-R	GGTCCAGGTTTCAGTTGCAA		
pmrB-F1	GCGAAAAGATTGGCAAATCG	659	This study
pmrB-R1	GGAAATGCTGGTGGTCATCTGA		
pmrB-F2	CCCTGAATCAGTTGGTTTC	714	This study
pmrB-R2	ATCAATGGGTGCTGACGTT		
mgrB-F	ACCACCTCAAAGAGAAGGCGTT	347	This study
mgrB-R	GGCGTGATTTTGACACGAACAC		
phoP-F	GAGCGTCAGACTACTATCGA	912	This study
phoP-R	GTTTTCCCATCTCGCCAGCA		
phoQ-F	CCACAGGACGTCATCACCA	1,594	This study
phoQ-R	GCAGGTGTCTGACAGGGATT		
mcr-1-F	CGGTCAGTCCGTTTGTTC	309	Liu et al., 2016
mcr-1-R	CTTGGTCGGTCTGTAGGG		
mcr-2-F	TGTTGCTTGTGCCGATTGGA	567	Xavier et al., 2016
mcr-2-R	AGATGGTATTGTTGGTTGCTG		
mcr-3-F	TTGGCACTGTATTTTGCATTT	542	Yin et al., 2017
mcr-3-R	TTAACGAAATTGGCTGGAACA		
mcr-4-F	ATTGGGATAGTCGCCTTTTT	487	Carattoli et al., 2017
mcr-4-R	TTACAGCCAGAATCATTATCA		
RT-qPCR			
pmrC-F	CTCTCGCCTCGTTCCTGAA	140	This study
pmrC-R	CGGAGTGGTGTCGAGGATA		
pmrK-F	GGTGTATGCGATTGGCACCTA	132	This study
pmrK-R	AGCAGCACGTAGCCCAGTAT		
rpsl-F	CCGTGGCGGTCGTGTTAAAGA	109	Cannatelli et al., 2013
rpsl-R	GCCGTACTTGGAGCGAGCCTG		
mgrB-ext-F	AAGGCGTTCATTCTACCACC	253	Cannatelli et al., 2013
mgrB-ext-R	TTAAGAAGGCCGTGCTATCC		
pmrB-ext-F	ACCTACGCGAAAAGATTGGC	1,274	Jayol et al., 2014
pmrB-ext-R	GATGAGGATAGCGCCCATGC		

### Transcriptional Analysis of *pmrK* and *pmrC* by RT-qPCR

To investigate whether colistin resistance is associated with upregulation of *pmrCAB* and *pmrHFIJKLM* operons, the expression level of genes encoding for LPS modifying enzymes including the *pmrK* (encoding for L-Ara4N transferase) and the *pmrC* (encoding for PEtN transferase) was evaluated. To this end, three col-S and 20 col-R KP isolates were grown to the mid-logarithmic phase of growth in LB broth medium and cells were harvested at an optical density of 1 at 600 nm (OD600). Bacterial cell wall was broken by several freeze/thaw cycles and total RNA was extracted using a GeneAll RiboEx Total RNA extraction kit (GeneAll Biotechnology, Seoul, South Korea). To remove genomic DNA contamination, total RNA was digested by RNase-free DNase I (Takara Biotechnology, Dalian, China) and then purified according to the manufacturer’s instructions. First-strand cDNA was synthesized from 1 μg of total RNA using Revert Aid first strand cDNA synthesis kit (Thermo Scientific). Real-time PCR amplification was performed using a Power SYBR green PCR master mix (Applied Biosystems) on a Eco Real-Time PCR system (Illumina) under the following conditions: 1 cycle of 95°C for 10 min, 45 cycles of 95°C for 15 s, 60°C for 30 s and 72°C for 25 s. Melt curve analyses were performed after each run to ensure single amplicon production. The Relative gene expression levels were calculated using the 2^-ΔΔC_T_^ formula with *rpsL* gene as internal control. Oligonucleotide sequences used for amplification of *pmrK, pmrC*, and *rpsL* fragments are listed in **Table 1**.

### Complementation Assays

To determine whether alterations in the *pmrB* and *mgrB* were mediating colistin resistance, complementation assay was performed. The full length *mgrB* and *pmrB* genes from a col-S KP isolate were amplified by PCR using primers pmrB-ext-F, pmrB-ext-R, mgrB-ext-F, and mgrB-ext-R (**Table 1**). The amplified fragments were cloned in to pCR-Blunt II-TOPO vector by Zero blunt TOPO PCR cloning kit (Invitrogen). The authenticity of the cloned fragments was confirmed by sequencing. The resulting pTOPO-mgrB and pTOPO-pmrB plasmids were introduced in to *Escherichia coli* TOP 10 by electroporation strategy and transformants were selected by overnight incubation at Muller–Hinton agar containing 50 μg/ml kanamycin. Plasmid purification was done by QIAprep Spin Miniprep Kit (Qiagen). Extracted plasmids were separately introduced to col-R KP isolates and transformants were selected on MHA plates supplemented with 250 μg/ml of zeocin. Overnight cultures of transformed col-R KP isolates were grown in MHB supplemented with 250 μg/ml zeocin, washed and resuspended in MHB and used for colistin MIC measurement. Col-S isolate KP51 was used as control.

### Nucleotide Sequence Accession Numbers

The nucleotide sequences of the wild-type *phoP, phoQ, pmrA, pmrB*, and *mgrB* genes have been deposited at GenBank nucleotide sequence database under the accession numbers MG243705 to MG243721, MF431844, and MF431845. Also nucleotide sequences of the altered *pmrB* and mutated/disrupted *mgrB* genes have been deposited under GenBank accession numbers MF431841 to MF431843 and MF431836 to MF431840, respectively.

## Results

### Bacterial Genotyping and Molecular Determinants of Colistin Resistance

**FIGURE 1:**
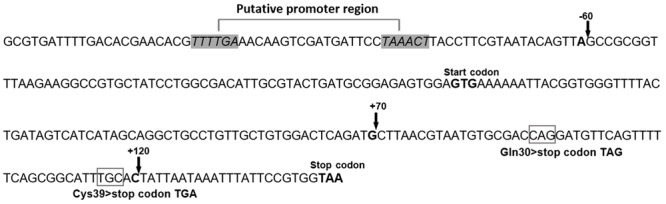
*mgrB* alterations mediated by insertion of IS elements and mutations. The arrow and open box indicate the target site for insertion of IS elements and amino acid changes, respectively.

A total of 20 col-R KP clinical isolates were studied to identify underlying resistance mechanisms. PFGE analyses divided the isolates into 17 PFGE patterns (pulsotypes) including A (KP17 and KP15), B1 (KP03), B2 (KP14), C1 (KP13), C2 (KP12), C3 (KP16), D1 (KP04), D2 (KP09), D3 (KP07), D4 (KP01), D5 (KP10), E (KP19 and KP20), F1 (KP08), F2 (KP06), G (KP05), H1 (KP02) and H2 (KP11). PFGE could not yield DNA fingerprints for isolate KP18 and therefore this isolate could not be genotyped. Isolates in types A and E were indistinguishable, and others were closely related (data not shown). To assess the role of plasmid encoded resistance genes, *mcr-1, mcr-2, mcr-3*, and *mcr-4*, PCR was carried out using genomic DNA obtained from 20 col-R, three col-S KP clinical isolates and *K. pneumoniae* ATCC 700603. *mcr* genes were not detected in any of the studied isolates indicating that chromosomally mediated mechanisms might play a prominent role in resistance occurrence. All col-R isolates revealed wild type *pmrA, phoP*, and *phoQ* genes. Fifteen col-R KP isolates (75%) exhibited alterations in the coding or putative promoter region of the *mgrB*. Two types of *mgrB* alterations were observed: premature termination due to nonsense mutations and insertion of IS elements (**Table 2**). In isolates characterized with *mgrB* premature termination, CAG (codon 30, glutamine) and TGC (codon39, cysteine) converted to TAG and TGA, respectively, creating a premature stop codon within the coding region of *mgrB* (**Figure 1**). These alterations resulted in production of a truncated and most likely non-functional 29 and 38 amino acid long proteins, respectively, instead of a wild-type protein with 47 amino acids. IS elements involved in the *mgrB* disruption belonged to IS*1*-like (768 bp) and IS*5*-like families (1,056 bp) which inserted in different positions of the *mgrB* gene. While in five isolates IS elements targeted coding region of the *mgrB* (positions +70 and +120), in one isolate IS element was found to be inserted at position -60 which was located between protein start codon and putative promoter region. The latter alteration disrupted the promoter and consequently transcription of the *mgrB* gene (**Figure 1**). Sequence analysis of the *pmrB* gene revealed that 19 of 20 col-R KP strains (95%) had a point mutation in the coding region of the *pmrB* including Leu213Met (CTG > ATG), Ala246Thr (GCC > ACC), and Arg256Gly (CGC > GGC) substitutions (**Table 2**). All col-S KP isolates revealed a wild-type *pmrA, pmrB, mgrB, phoP*, and *phoQ* sequences.

**Table 2:** Phenotypic and genotypic characteristics of the studied *K. pneumoniae* strains.

Isolate	Year, ward, and source of isolation	MIC (μg/ml)	*mgrB* sequence	*pmrB* sequence	Relative expression level	
					*pmrK*	*pmrC*
KP13	2016-Neurology-Wound	128	Insertional inactivation by IS*1*-like (+120)	A246T	3.83 ± 1.01	–^a^
KP12	2016-Emergency-Blood	64	Insertional inactivation by IS*1*-like (+120)	A246T	4.12 ± 0.05	–^a^
KP16	2016-ICU-Wound	128	Insertional inactivation by IS*1*-like (+120)	A246T	3.01 ± 0.02	–^a^
KP01	2015-ICU-Blood	128	Premature termination by nonsense mutation at nt88	L213M	13.04 ± 0.5	1.87 ± 0.18
KP04	2015-ICU-Blood	>128	Premature termination by nonsense mutation at nt88	L213M	7.08 ± 0.25	3.5 ± 0.07
KP07	2015-ICU-Tracheal aspirate	>128	Premature termination by nonsense mutation at nt88	L213M	26.91 ± 2.68	–^a^
KP08	2016-ICU-Blood	128	Premature termination by nonsense mutation at nt88	L213M	2.1 ± 0.77	–^a^
KP09	2015-SICU-Tracheal aspirate	>128	Premature termination by nonsense mutation at nt88	L213M	34.47 ± 1.0	9 ± 2.1
KP10	2016-ICU-Tracheal aspirate	>128	Premature termination by nonsense mutation at nt88	L213M	3.17 ± 0.03	3.66 ± 1.08
KP18	2017-Internal medicine-Blood	>128	Premature termination by nonsense mutation at nt88	L213M	1.97 ± 0.04	–^a^
KP17	2016-Urology-Tracheal aspirate	128	Premature termination by nonsense mutation at nt117	A246T	3.96 ± 0.04	–^a^
KP15	2016-ICU-Blood	128	premature termination by nonsense mutation at nt117	A246T	1.98 ± 0.12	–^a^
KP02	2015-SICU-Urine	32	Insertional inactivation by IS*5*-like (+70)	R256G	2.56 ± 0.14	3.26 ± 0.2
KP11	2016-Internal medicine-Wound	128	Insertional inactivation by IS*5*-like (+70)	R256G	77 ± 1.50	15.4 ± 3.9
KP06	2016-Neurology-Tracheal aspirate	64	Insertional inactivation by IS*1*-like at promoter region (-60)	WT	11.69 ± 2.59	9.15 ± 1.5
KP03	2015-SICU-Blood	128	WT^b^	R256G	64.36 ± 2.6	6.9 ± 0.83
KP05	2015-Urology-Urine	8	WT	R256G	24.3 ± 0.23	2.79 ± 0.15
KP14	2016-Emergency-Urine	128	WT	R256G	76.07 ± 6.2	7.06 ± 2.1
KP51	2016-CCU-Urine	0.5	WT	WT	–^a^	–^a^
KP106	2016-Outpatient clinic-Urine	0.5	WT	WT	–^a^	–^a^
KP109	2016-Orthopedics-Urine	0.25	WT	WT	–^a^	–^a^
KP19	2017-ICU-Blood	128	WT	A246T	2.2 ± 0.23	1.67 ± 0.33
KP20	2017-ICU-Blood	128	WT	A246T	2.5 ± 0.7	1.7 ± 0.53

*^a^Unaltered expression levels compared to col-S isolates. ^b^WT, wild-type.*

### Colistin Resistance Is Associated with Overexpression of Operons Encoding for LPS Modifying Proteins

To examine the association between colistin resistance and *pmrHFIJKLM* and *pmrCAB* expression, the transcription levels of *pmrK* and *pmrC* genes encoding proteins implicated in decoration of LPS with L-Ara4N and PEtN, respectively, were analyzed by RT-qPCR method. In general, all col-R KP isolates showed an elevation in the transcription level of *pmrK* from 1.97- to 77-fold compared to the levels obtained for our laboratory col-S wild-type strains. Moreover, 11 (55%) col-R KP isolates were found to have overexpressed *pmrC* gene from 1.67- to 15.4-fold (**Table 2**). Our results indicated that colistin resistance significantly correlated with *pmrHFIJKLM* or *pmrCAB* upregulation. Elevated expression of studied genes was irrespective of the absence or presence of colistin (data not shown). Our results indicate that the incorporation of both aminoarabinose and PEtN into lipid A is responsible for colistin resistance with the latter being inferior to that of L-Ara4N substitution.

### Complementation Experiments

Expression of wild type MgrB in isolates characterized with altered *mgrB* restored susceptibility to colistin and reduced the MIC values to levels obtained for col-S isolates (**Table 3**). However, complementation with wild type PmrB did not alter colistin susceptibility in col-R isolates harboring mutated *pmrB*. This indicates that colistin resistance is not related to these *pmrB* alterations and modifications in loci other than *pmrB* might be involved in colistin resistance in these isolates.

**Table 3:** MICs of colistin in *K. pneumoniae* isolates before and after complementation.

Isolate	*mgrB* genotype	*pmrB* genotype	Complemented gene	MIC befor complementation	MIC after complementation
KP06	IS insertion at -60	WT	*mgrB*	64	1
KP12	IS insertion at +120	A246T	*mgrB*	64	1
KP02	IS insertion at +70	R256G	*mgrB*	32	1
KP01	Q30stop	L213M	*mgrB*	128	2
			*pmrB*		128
KP17	C39stop	A246T	*mgrB*	128	2
KP03	WT	R256G	*pmrB*	128	128
KP19	WT	A246T	*pmrB*	128	128

## Discussion

Colistin has recently regained significant interest constituting the major part of therapeutic regimen for management of severe infections caused by CRE (Morrill et al., 2015). Despite the scarcity of colistin resistance, this anti-CRE magic bullet has begun to wane as a consequence of frequent use in clinical settings and subsequent resistance development. In our study, plasmid encoded *mcr-1, mcr-2, mcr-3, and mcr-4* genes were not detected in any of the col-R KP isolates demonstrating that resistance is mediated by chromosomally encoded mechanisms. Of 20 col-R KP isolates, 15 (75%) were identified as having an inactivated MgrB mediated by insertion of IS elements or nonsense mutations. MgrB, a small transmembrane protein with 47 amino acids mediates potent negative feedback on the PhoQ/PhoP regulatory system. It is hypothesized that critical alterations within *mgrB* found in the current study including disruption of promoter or coding region result in gene silencing or production of truncated MgrB. In fact, MgrB inactivation by any of these events results in PhoP/PhoQ activation which in turn activates the PmrA response regulator. Complementation with wild type MgrB protein restored colistin susceptibility in all col-R KP isolates with *mgrB* modifications. This verifies the resistance conferring role of *mgrB* alterations in the studied isolates. Poirel et al. (2015) demonstrated that insertion of IS elements (IS*5*-like, IS*Kpn13* IS*Kpn14*, IS*10R*) and Q30stop and C28stop substitutions in *mgrB* were involved in resistance to colistin in *K. pneumoniae* isolates determined by complementation assays. The Q30stop substitution has been found by several other studies (Olaitan et al., 2014a; Aires et al., 2016) reinforcing the hypothesis that position +88 in the *mgrB* (codon 30 in protein) is a critical region which is prone to mutate upon resistance emergence. Also, Cannatelli et al. (2014b) reported that *mgrB* alterations, including insertion of IS*5*-like, IS*1*-like, or IS*Kpn14* elements, non-silent point mutations, and small intragenic deletions were mediating colistin resistance in 53% of studied col-R KP isolates. All together three substitutions were found in the *pmrB* including L213M, A246T, and R256G. Complementation with wild type *pmrB* gene was not able to restore colistin susceptibility in isolates characterized with these *pmrB* alterations. Similarly, Cheng et al. (2015) showed that R256G substitution found in col-R KP isolates did not contribute to colistin resistance determined by site-directed mutagenesis method. Also, Aires et al. (2016) suggested that A246T mutation found in the *pmrB* was not related to colistin resistance. This was because they had found this alteration in colistin susceptible isolates as well. Therefore, mechanisms of colistin resistance were not fully unraveled in five isolates characterized with *pmrB* mutations alone. A recent study showed that alterations in the newly described TCRS, CrrAB mediate colistin resistance in *K. pneumoniae* (Wright et al., 2015). Cheng et al. (2015) found R256G replacement in the *pmrB* as the only genetic alteration among six studied genes including *mgrB, pmrA, pmrB, phoP, phoQ*, and *pmrD* in 8 of 26 col-R KP isolates. All of these eight isolates had overexpressed *pmrHFIJKLM* operon. In an attempt to determine the exact mechanisms of decreased susceptibility to colistin in these isolates, the same authors found mutations in CrrAB TCRS. The mutations were found to be associated with overexpression of *pmrHFIJKLM* and *pmrCAB* operons in these isolates (Cheng et al., 2016). Therefore, it is tempting to speculate that genetic modifications in systems other than PmrA/PmrB and PhoP/PhoQ such as CrrAB might contribute to colistin resistance in these five isolates.

Colistin resistance has been found to be associated with upregulation of *pmrHFIJKLM* and *pmrCAB* operons which result in addition of L-Ara4N and PEtN, to LPS, respectively. Generally, either L-Ara4N and/or PEtN can be added to the 4-phosphate and 1-phosphate of lipid A and both modifications decrease the anionic charge of LPS (Zhou et al., 2001; Lee et al., 2004; Tamayo et al., 2005). However, it has been demonstrated that the LPS modification by L-Ara4N confers a higher level of polymyxin resistance compared to PEtN modification (Tamayo et al., 2005). We found a significant association between colistin resistance and upregulation of *pmrHFIJKLM* and *pmrCAB* operons. While all isolates revealed an over-expressed *pmrK*, only 55% of isolates exhibited *pmrC* upregulation implying that *pmrC*-mediated LPS modification was inferior to that of *pmrK* with regard to colistin resistance. However, one isolate (KP02) revealed partially higher expression level for *pmrC* (3.2-fold) compared to *pmrK* gene (2.5-fold). Jayol et al. (2014) reported that Thr157Pro substitution in PmrB was responsible for the overexpression of *pmrCAB* and *pmrHFIJKLM* operons with the *pmrC* being more overexpressed (170-fold) compared to *pmrK* (40-fold). We also observed that strains with the same mutational profile and colistin MIC values revealed distinct expression level/profile of *pmrK* or *pmrC* genes. This can be explained by the fact that determinants other than PmrB/MgrB (such as CrrAB TCRS) might contribute to regulation of operons involved in the LPS modification. Also, different MIC values between the isolates with the same genetic alterations in the studied loci can be attributed to alterations in other unknown loci or involvement of other resistance mechanisms such as increased amount of capsular polysaccharide or efflux pumps (Olaitan et al., 2014b).

In summary, we found that MgrB alterations play major role in colistin resistance in *K. pneumoniae* isolates studied in the current work. We also found that, *pmrK* operon and therefore LPS modification with L-Ara4N plays prominent role in resistance conferring followed by *pmrC* operon and PEtN modification. This is the first report about molecular mechanisms of colistin resistance in *K. pneumoniae* isolates from Iran. Evaluating the role of CrrAB system in mediating colistin resistance will be the next step of this ongoing work to provide a comprehensive understanding of mechanisms involved in colistin non-susceptibility in *K. pneumoniae* isolates. In addition, multilocus sequence typing should be performed to determine whether these Iranian col-R KP isolates belong to predominant sequence types in Iran or to those, which are common in the world.

## Author Contributions

MH is the corresponding author who designed the experiments, analyzed the experiment data, and wrote the manuscript. AJ performed the experiments, analyzed the experiment data, and drafted the manuscript. JM and ZJ contributed to performance of complementation assays and PFGE analysis respectively. MF designed the experiments and wrote the manuscript. EB participated in the design and performance of RNA extraction and RT-qPCR and editing the manuscript.

## Conflict of Interest Statement

The authors declare that the research was conducted in the absence of any commercial or financial relationships that could be construed as a potential conflict of interest.

## Footnotes

1 http://www.eucast.org/

## Abbreviations

col-R KP: colistin resistant *K. pneumoniae*
col-S KP: colistin susceptible *K. pneumoniae*
CRE: carbapenem-resistant *Enterobacteriaceae*
GNB: Gram-negative bacteria
L-Ara4N: 4-amino-4-deoxy-L-arabinose
MDR: multidrug-resistant
PEtN: phosphoethanolamine

## References

[B1] Aires C. A. M. Pereira P. S. Asensi M. D. Carvalho-Assef A. P. D. A. (2016). *MgrB* mutations mediating polymyxin B resistance in *Klebsiella pneumoniae* isolates from rectal surveillance swabs in Brazil. *Antimicrob. Agents Chemother.* 60 6969–6972. 10.1128/AAC.01456-16 27620478 PMC5075120

[B2] Cannatelli A. D’Andrea M. M. Giani T. Di Pilato V. Arena F. Ambretti S. (2013). In vivo emergence of colistin resistance in *Klebsiella pneumoniae* producing KPC-type carbapenemases mediated by insertional inactivation of the PhoQ/PhoP *mgrB* regulator. *Antimicrob. Agents Chemother.* 7 5521–5526. 10.1128/AAC.01480-13 23979739 PMC3811314

[B3] Cannatelli A. Di Pilato V. Giani T. Arena F. Ambretti S. Gaibani P. (2014a). *In Vivo* evolution to colistin resistance by PmrB sensor kinase mutation in KPC-producing *Klebsiella pneumoniae* is associated with low-dosage colistin treatment. *Antimicrob. Agents Chemother.* 58 4399–4403. 10.1128/AAC.02555-14 24841267 PMC4136067

[B4] Cannatelli A. Giani T. D’Andrea M. M. Di Pilato V. Arena F. Conte V. (2014b). MgrB inactivation is a common mechanism of colistin resistance in KPC-producing *Klebsiella pneumoniae* of clinical origin. *Antimicrob. Agents Chemother.* 58 5696–5703. 10.1128/AAC.03110-14 25022583 PMC4187966

[B5] Carattoli A. Villa L. Feudi C. Curcio L. Orsini S. Luppi A. (2017). Novel plasmid-mediated colistin resistance mcr-4 gene in *Salmonella* and *Escherichia coli*, Italy 2013 Spain and Belgium, 2015 to 2016. *Euro Surveill.* 22:30589. 10.2807/1560-7917.ES.2017.22.31.30589 28797329 PMC5553062

[B6] Cheng Y.-H. Lin T.-L. Lin Y.-T. Wang J.-T. (2016). Amino acid substitutions of CrrB responsible for resistance to colistin through CrrC in *Klebsiella pneumoniae*. *Antimicrob. Agents Chemother.* 60 3709–3716. 10.1128/AAC.00009-16 27067316 PMC4879426

[B7] Cheng Y. H. Lin T. L. Pan Y. J. Wang Y. P. Lin Y. T. Wang J. T. (2015). Colistin resistance mechanisms in *Klebsiella pneumoniae* strains from Taiwan. *Antimicrob. Agents Chemother.* 59 2909–2913. 10.1128/AAC.04763-14 25691646 PMC4394772

[B8] Clinical and Laboratory Standards Institute (2014). *Performance Standards for Antimicrobial Susceptibility Testing: Twenty-Fourth Informational Supplement, M100-S24.* Wayne, PA: Clinical and Laboratory Standards Institute

[B9] Deris Z. Z. Swarbrick J. D. Roberts K. D. Azad M. A. Akter J. Horne A. S. (2014). Probing the penetration of antimicrobial polymyxin lipopeptides into gram-negative bacteria. *Bioconjug. Chem.* 25 750–760. 10.1021/bc500094d 24635310 PMC3993906

[B10] Gu D. X. Huang Y. L. Ma J. H. Zhou H. W. Fang Y. Cai J. C. (2016). Detection of colistin resistance gene mcr-1 in hypervirulent *Klebsiella pneumoniae* and *Escherichia coli* isolates from an infant with diarrhea in China. *Antimicrob. Agents Chemother.* 60 5099–5100. 10.1128/AAC.00476-16 27270278 PMC4958155

[B11] Gupta S. Govil D. Kakar P. N. Prakash O. Arora D. Das S. (2009). Colistin and polymyxin B: a re-emergence. *Indian J. Crit. Care Med.* 13 49–53. 10.4103/0972-5229.56048 19881183 PMC2772240

[B12] Jayol A. Poirel L. Brink A. Villegas M.-V. Yilmaz M. Nordmann P. (2014). Resistance to colistin associated with a single amino acid change in protein PmrB among *Klebsiella pneumoniae* isolates of worldwide origin. *Antimicrob. Agents Chemother.* 58 4762–4766. 10.1128/AAC.00084-14 24914122 PMC4136042

[B13] Li J. Nation R. L. Turnidge J. D. Milne R. W. Coulthard K. Rayner C. R. (2006). Colistin: the re-emerging antibiotic for multidrug-resistant gram-negative bacterial infections. *Lancet Infect. Dis.* 6 589–601. 10.1016/S1473-3099(06)70580-1 16931410

[B14] Lee H. Hsu F.-F. Turk J. Groisman E. A. (2004). The PmrA-regulated *pmrC* gene mediates phosphoethanolamine modification of lipid and polymyxin resistance in *Salmonella enterica*. *J. Bacteriol.* 186 4124–4133. 10.1128/JB.186.13.4124-4133.2004 15205413 PMC421605

[B15] Liu Y-Y. Wang Y. Walsh T. R. Yi L.-X. Zhang R. Spencer J. (2016). Emergence of plasmid-mediated colistin resistance mechanism MCR-1 in animals and human beings in China: a microbiological and molecular biological study. *Lancet Infect. Dis.* 16 161–168. 10.1016/S1473-3099(15)00424-7 26603172

[B16] Marinelli F. Genilloud O. (2013). *Antimicrobials: New and Old Molecules in the Fight Against Multi-resistant Bacteria.* New York, NY: Springer Science and Business Media.

[B17] Morrill H. J. Pogue J. M. Kaye K. S. LaPlante K. L. (2015). Treatment Options for carbapenem-resistant enterobacteriaceae infections. *Open Forum Infect. Dis.* 2:ofv050. 10.1093/ofid/ofv050 26125030 PMC4462593

[B18] Olaitan A. O. Diene S. M. Kempf M. Berrazeg M. Bakour S. Gupta S. K. (2014a). Worldwide emergence of colistin resistance in *Klebsiella pneumoniae* from healthy humans and patients in Lao PDR, Thailand, Israel, Nigeria and France owing to inactivation of the PhoP/PhoQ regulator *mgrB*: an epidemiological and molecular study. *Int. J. Antimicrob. Agents* 44 500–507. 10.1016/j.ijantimicag.2014.07.020 25264127

[B19] Olaitan A. O. Morand S. Rolain J.-M. (2014b). Mechanisms of polymyxin resistance: acquired and intrinsic resistance in bacteria. *Front. Microbiol.* 5:643 10.3389/fmicb.2014.00643 PMC4244539 25505462

[B20] Poirel L. Jayol A. Bontron S. Villegas M. V. Ozdamar M. Turkoglu S. (2015). The mgrB gene as a key target for acquired resistance to colistin in *Klebsiella pneumoniae*. *J. Antimicrob. Chemother.* 70 75–80. 10.1093/jac/dku323 25190723

[B21] Ribot E. M. Fair M. Gautom R. Cameron D. Hunter S. Swaminathan B. (2006). Standardization of pulsed-field gel electrophoresis protocols for the subtyping of *Escherichia coli* O157: H7 *Salmonella*, and *Shigella* for PulseNet. *Foodborne Pathog. Dis.* 3 59–67. 10.1089/fpd.2006.3.59 16602980

[B22] Tamayo R. Choudhury B. Septer A. Merighi M. Carlson R. Gunn J. (2005). Identification of *cptA*, a PmrA-regulated locus required for phosphoethanolamine modification of the *Salmonella enterica* serovar typhimurium lipopolysaccharide core. *J. Bacteriol.* 187 3391–3399. 10.1128/JB.187.10.3391-3399.2005 15866924 PMC1112023

[B23] Tenover F. C. Arbeit R. D. Goering R. V. Mickelsen P. A. Murray B. E. Persing D. H. (1995). Interpreting chromosomal DNA restriction patterns produced by pulsed-field gel electrophoresis: criteria for bacterial strain typing. *J. Clin. Microbiol.* 33 2233–2239. 7494007 10.1128/jcm.33.9.2233-2239.1995 PMC228385

[B24] Wright M. S. Suzuki Y. Jones M. B. Marshall S. H. Rudin S. D. Van Duin D. (2015). Genomic and transcriptomic analyses of colistin-resistant clinical isolates of *Klebsiella pneumoniae* reveal multiple pathways of resistance. *Antimicrob. Agents Chemother.* 59 536–543. 10.1128/AAC.04037-14 25385117 PMC4291396

[B25] Wiegand I. Hilpert K. Hancock R. E. (2008). Agar and broth dilution methods to determine the minimal inhibitory concentration of antimicrobial substances. *Nat. Protoc.* 3 163–175. 10.1038/nprot.2007.521 18274517

[B26] Xavier B. B. Lammens C. Ruhal R. Kumar-Singh S. Butaye P. Goossens H. (2016). Identification of a novel plasmid-mediated colistin-resistance gene, *mcr-2*, in *Escherichia coli*, Belgium, June 2016. *Euro Surveill.* 21:30280. 10.2807/1560-7917.ES.2016.21.27.30280 27416987

[B27] Yin W. Li H. Shen Y. Liu Z. Wang S. Shen Z. (2017). Novel plasmid-mediated colistin resistance gene *mcr-3* in *Escherichia coli*. *mBio.* 8:e00543-17. 10.1128/mBio.00543-17 28655818 PMC5487729

[B28] Zhou Z. Ribeiro A. A. Lin S. Cotter R. J. Miller S. I. Raetz C. R. (2001). Lipid a modifications in polymyxin-resistant *Salmonella typhimurium* PmrA-dependent 4-amino-4-deoxy-l-arabinose, and phosphoethanolamine incorporation. *J. Biol. Chem.* 276 43111–43121. 10.1074/jbc.M106960200 11535603

